# Patterns and Outcomes of Induction of Labour in Africa and Asia: A Secondary Analysis of the WHO Global Survey on Maternal and Neonatal Health

**DOI:** 10.1371/journal.pone.0065612

**Published:** 2013-06-03

**Authors:** Joshua P. Vogel, João Paulo Souza, A. Metin Gülmezoglu

**Affiliations:** 1 School of Population Health, Faculty of Medicine, Dentistry and Health Sciences, University of Western Australia, Perth, Western Australia, Australia; 2 UNDP/UNFPA/UNICEF/WHO/World Bank Special Programme of Research, Development and Research Training in Human Reproduction (HRP), Department of Reproductive Health and Research, World Health Organization, Geneva, Switzerland; Aga Khan University, Pakistan

## Abstract

**Background:**

Labour induction should be performed where benefit outweighs potential harm, however epidemiology of induction in lower-income countries is not well described. We used the WHO Global Survey dataset to describe the epidemiology and outcomes of labour induction in 192,538 deliveries in 253 facilities across 16 countries in Africa and Asia.

**Methods:**

Data was analyzed separately for Africa and Asia. Prevalence of indications, methods, success and characteristics associated with labour induction were determined. Multilevel logistic regression was used to determine the relationship between induction (with medical indication and elective) and maternal/perinatal outcomes.

**Results:**

Induction accounted for 4.4% (Africa) and 12.1% (Asia) of deliveries. Oxytocin alone was the most common method (45.9% and 37.5%) and success rates were generally over 80%. Medically indicated inductions were associated with increased adjusted odds of Apgar <7 at 5 minutes, low birthweight, NICU admission and fresh stillbirth in both regions. The odds of caesarean section in Africa were reduced (Adj OR 0.61, 95%CI 0.42–0.88). Elective induction was associated with increased adjusted odds of NICU (Africa) and ICU (Asia) admissions.

**Discussion:**

Induction was generally less common than in higher-income countries. Prostaglandin use was uncommon despite evidence supporting use. Induction for medical indications may be associated with poorer outcomes due to maternal baseline risks. Despite one-third of elective inductions occurring at <39 weeks, the risk of maternal, fetal and neonatal mortality was not elevated following elective inductions.

## Introduction

Induction of labour is indicated where the benefits to mother and/or fetus of discontinuing the pregnancy outweigh the risks of awaiting spontaneous onset of labour [1], [2]. However, induction of labour is not without risk. The World Health Organization (WHO) recommends induction be performed with a clear medical indication and when expected benefits outweigh potential harms [3]. Success of induction depends largely on cervical status; an unripe cervix conveys a lower likelihood of vaginal delivery [1]. Beyond 41 weeks of gestation, induction is associated with a small reduction in perinatal deaths and meconium aspiration syndrome [4]. Induction following premature rupture of membranes (PROM) has been shown to reduce chorioamnionitis, endometritis and neonatal ICU (NICU) admissions [5]. Other established indications include hypertensive disorders, chorioamnionitis, maternal medical complications, intra-uterine growth restriction, fetal death, vaginal bleeding, multiple pregnancy and isoimmunization [1], [3], [5].

Induction accounts for approximately 20% of deliveries in the UK and USA [1], [2] and rates have been rising steadily [6], [7]. This has been attributed to patient and physician factors, however elective induction rates are increasing disproportionately [8], [9], accounting for 10 to 30% of inductions in some countries [7]–[10]. Some studies of elective induction suggest higher rates of adverse outcomes, including prolonged first stage, failure to progress, intrapartum haemorrhage, admission to NICU and a higher incidence of assisted vaginal birth [2], [11]–[13]. Evidence from these higher-resource settings suggests that increased induction use is not associated with higher rates of caesarean section [8], [13], [14]. This has implications for both higher-resource settings where the caesarean section rate may be too high, but also low-resource settings with limited access to safe caesarean section [15].

The epidemiology of labour induction in lower-income countries is not as well described. While induction is generally not as widespread, some hospitals have rates comparable to higher-income countries [3]. Many countries have significant challenges in drug availability, healthcare staffing, obstetric and fetal monitoring facilities and access to safe caesarean section, complicating the availability, access and scale up of safe induction. Two previous analyses by Guerra and colleagues of labour induction in Latin America were based on WHO Global Survey (WHOGS) data and found an induction rate of 11.4%, of which 16.7% were elective [11], [16]. Premature rupture of membranes was the most frequent medical indication in all countries, and induction was associated with increased uterotonic use, perineal lacerations, hysterectomy, ICU & NICU admission, longer hospital stays, greater anaesthesia/analgesia requirements during labour, lower Apgar scores and delayed commencement of breastfeeding [16]. However, elective induction was the most common overall indication and was associated with increased uterotonic use, ICU admission, hysterectomy, greater anaesthesia/analgesia use and delayed commencement of breastfeeding [11]. The WHOGS secondary analysis by Fawole and colleagues determined that African countries had a high rate (66.0–80.2%) of unmet need for labour induction [17].

Describing the pattern of labour induction in lower-income countries is a crucial step in its safe and proper application. We aimed to determine the prevalence, indications, methods and success of induction of labour (with and without medical indication) in 253 facilities across 16 countries in Africa and Asia. We also used multilevel logistic regression to examine the effect of induction of labour on maternal and neonatal outcomes.

## Methods

The WHO Global Survey on Maternal and Perinatal Health (WHOGS) was a multi-country, cross-sectional survey of all deliveries occurring in participating institutions over a two or three-month period (depending on annual volume of deliveries). The methodological details have been published previously [18], [19]. A stratified, multistage sampling design was used to obtain a global sample of countries and health institutions. Countries in WHO regions were grouped according to adult and child mortality and countries were randomly selected from each sub-region (probability proportional to population). Twenty-four countries were willing and able to participate. Using the capital city and two randomly selected provinces (probability proportional to population), seven institutions were randomly selected (probability proportional to births per year) from a census of institutions with over 1,000 births annually and capacity to perform caesarean section. Trained data collectors reviewed patient records to complete the individual data forms for all deliveries occurring in the study period. Deliveries occurring outside the participating institutions were not recorded. The study was conducted over 2004–2005 (Africa and Latin America) and 2007–2008 (Asia).

The WHOGS dataset contains data on over 290,000 deliveries in 373 institutions across Africa, Latin America and Asia. Induction in Latin America has been previously explored [1], [2], [11], [16], thus this analysis considered facility deliveries in the seven African countries (Algeria, Angola, DR Congo, Kenya, Niger, Nigeria and Uganda) and eight Asian countries (Cambodia, China, India, Japan, Nepal, Philippines, Sri Lanka, Thailand and Viet Nam). We included only women who underwent spontaneous or induced labour and excluded those who did not undergo labour.

Maternal outcomes of interest were delivery by caesarean section following induction (ie: failure of induction), maternal death, blood transfusion, 3^rd^ or 4^th^ degree perineal laceration, hysterectomy, admission to an intensive care unit (ICU), postpartum hospital stay >7 days and commencement of breastfeeding post-delivery (by 24 hours and by day 7). Perinatal outcomes of interest were Apgar score <7 at 5 minutes, low birthweight (<2500 g), admission to NICU, “fresh” stillbirth (as a proxy for intrapartum stillbirth) and early neonatal death. Maternal and early neonatal deaths occurring before discharge or day 7 postpartum were recorded, whereas deaths occurring after discharge, day 7 postpartum or during a postpartum readmission were not captured.

Data from the two regions were analyzed and reported separately throughout. Mothers were stratified into three groups – spontaneous labour, induction with a medical indication and elective induction. We defined medical indications using criteria in the WHOGS individual data form, namely one or more of: fetal death, suspected fetal growth impairment, fetal distress, multiple pregnancy, prelabour rupture of membranes (PROM), chorioamnionitis, vaginal bleeding, pre-eclampsia/eclampsia, gestational age of 41 or more completed weeks or any other obstetric or maternal medical complication. Elective inductions were defined as inductions recorded as elective and/or by maternal request only. Maternal, neonatal and facility characteristics were compared across these three groups using percentages and overall chi square tests of significance, adjusted for survey design (using the Complex Samples module in SPSS, with countries as strata and facilities as clusters). Patterns, indications and success of induction were reported as percentages at the regional and country level. We determined crude and adjusted odds ratios between these groups and selected outcomes (spontaneous labour was the reference group). Adjusted odds ratios were obtained using generalized linear mixed models within each region. All maternal models were adjusted for maternal age, marital status, years of maternal education, parity, number of antenatal care visits, maternal height (as a proxy for maternal undernutrition), mode of delivery (vaginal or caesarean section) and previous caesarean section. These models were also adjusted for medical and obstetric conditions (as binary variables, unless otherwise stated): PROM, pre-eclampsia, chronic hypertension, pregnancy-induced hypertension, cardiac/renal disease, respiratory disease, suspected fetal growth impairment, diabetes, severe anaemia (Hb<7 g/L), vaginal bleeding in the second half of pregnancy, HIV, malaria, gestational age (<37 weeks, 37–42 weeks, >42 weeks), urinary infection/pyelonephritis, fetal presentation (cephalic, breech or other) and attendant at delivery (doctor, midwife/nurse, other). The model for delivery by caesarean section (failure of induction) did not include mode of delivery as a confounder. Perinatal outcomes were adjusted for the same confounders, as well as infant sex and congenital malformation. All models included facility as a random effect variable to account for clustering; missing values were excluded. All analysis was performed using SPSS 20 [3], [20], data was reported at the regional level (Supplementary Tables contain country-level data). Clearance from all Ministries of Health of participating countries, WHO Ethics Review Committee and relevant local ethical boards was obtained.

## Results

**Figure 1:**
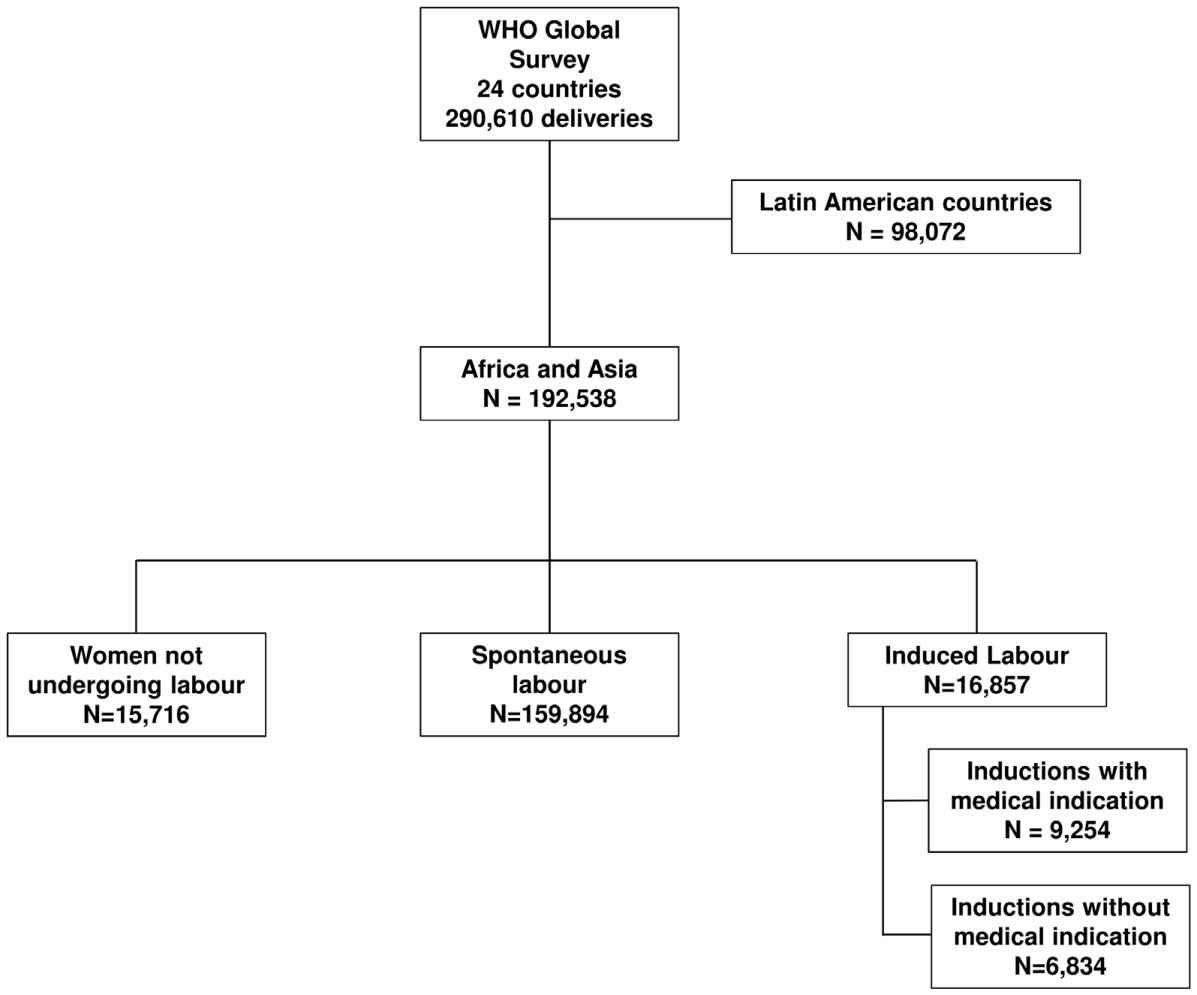
Study flow chart.

We analyzed data from 192,538 deliveries in 253 facilities across 16 countries in Africa (n = 83,437) and Asia (n = 109,101) (Figure 1). There were significant differences between mode of labour and maternal, neonatal and facility characteristics, however these varied by region (Tables 1 and 2). In both regions, ten or more years of education, nulliparity and 4 or more antenatal care visits were more frequent in women undergoing any form of induction compared to spontaneous labour, as were most medical conditions. Inductions were more frequently in urban and tertiary facilities in Africa, whereas this was not significantly different in Asia.

**Table 1:** Maternal and perinatal characteristics by labour status in Africa and Asia.

	AFRICA				ASIA			
	Induction with medical indication	Elective induction	Spontaneous labour	Adjusted chi squareP value^a^	Induction with medical indication	Elective induction	Spontaneous labour	Adjusted chi squareP value^a^
	N, (%)	N, (%)	N, (%)		N, (%)	N, (%)	N, (%)	
**TOTAL**	**3,053**	**564**	**76,965**		**6,201**	**6,270**	**82,929**	
**Maternal age (years)** b								
<18	99 (3.2)	15 (2.7)	3900 (5.1)	<0.001	67 (1.1)	98 (1.6)	1400 (1.7)	0.319
18–35	2,488 (81.5)	493 (87.4)	65165 (84.7)		5,788 (93.3)	5,747 (91.7)	76741 (92.5)	
>35	452 (14.8)	452 (14.8)	7324 (9.5)		345 (5.6)	423 (6.7)	4773 (5.8)	
**Marital Status** b								
Married	2,705 (88.6)	510 (90.4)	66653 (86.6)	0.282	5,947 (95.9)	6,178 (98.5)	76867 (92.7)	<0.001
**Education (years)**								
Nil	273 (8.9)	46 (8.2)	10749 (14.0)	<0.001	533 (8.6)	298 (4.8)	7719 (9.3)	0.003
1–4	156 (5.1)	17 (3.0)	4613 (6.0)		287 (4.6)	172 (2.7)	4393 (5.3)	
5–9	980 (32.1)	162 (28.7)	28303 (36.8)		1,818 (29.3)	1,405 (22.4)	29696 (35.8)	
> = 10	1,441 (47.2)	320 (56.7)	25994 (33.8)		3,385 (54.6)	4,248 (67.8)	39484 (47.6)	
Missing	203 (6.6)	19 (3.4)	7306 (9.5)		178 (2.9)	147 (2.3)	1637 (2.0)	
**Parity**								
Nil	1,245 (40.8)	210 (37.2)	25946 (33.7)	0.005	3,888 (62.7)	3,324 (53.0)	41391 (49.9)	<0.001
1 or 2	994 (32.6)	204 (36.2)	29669 (38.5)		2,079 (33.5)	2,671 (42.6)	36270 (43.7)	
> = 3	769 (25.2)	142 (25.2)	20701 (26.9)		232 (3.7)	274 (4.4)	5210 (6.3)	
Missing	45 (1.5)	8 (1.4)	649 (0.8)		2 (0.0)	1 (0.0)	58 (0.1)	
**Antenatal care**								
Nil	184 (6.0)	18 (3.2)	4084 (5.3)	<0.001	342 (5.5)	52 (0.8)	4350 (5.2)	<0.001
1 to 3	591 (19.4)	117 (20.7)	26912 (35.0)		1,473 (23.8)	548 (8.7)	25948 (31.1)	
> = 4	1,939 (63.5)	340 (60.3)	35258 (45.8)		4,308 (69.5)	5,590 (89.2)	51795 (62.5)	
Missing	339 (11.1)	89 (15.8)	10711 (13.9)		78 (1.3)	80 (1.3)	836 (1.0)	
**Maternal Height**								
<145 cm	42 (1.4)	16 (2.8)	970 (1.3)	0.168	247 (4.0)	269 (4.3)	2659 (3.2)	0.174
145–149.9 cm	76 (2.5)	30 (5.3)	2162 (2.8)		593 (9.6)	845 (13.5)	2659 (3.2)	
150–154.9 cm	300 (9.8)	79 (14.0)	8099 (10.5)		2,267 (36.6)	2,035 (32.5)	29363 (35.4)	
> = 155 cm	1,994 (65.3)	307 (54.4)	45176 (58.7)		2,994 (48.3)	3,067 (48.9)	40005 (48.2)	
Missing	641 (21.0)	132 (23.4)	20558 (26.7)		100 (1.6)	54 (0.9)	2498 (3.0)	
**Caesarean in previous pregnancy**	49 (1.6)	30 (5.3)	2938 (3.8)	0.085	100 (1.6)	101 (1.6)	4265 (5.1)	<0.001
Missing	9 (0.3)	1 (0.2)	172 (0.2)		3,366 (54.3)	2,923 (46.6)	35943 (43.3)	
**Prelabour rupture of membranes** b	834 (27.3)	32 (5.7)	4625 (6.0)	<0.001	2,191 (35.3)	282 (4.5)	9453 (11.4)	<0.001
**Chronic hypertension** b	39 (1.3)	3 (0.5)	261 (0.3)	<0.001	49 (0.8)	11 (0.2)	359 (0.4)	0.003
**Pregnancy-induced hypertension** b	264 (8.6)	29 (5.1)	1574 (2.0)	<0.001	588 (9.5)	227 (3.6)	2378 (2.9)	<0.001
**Pre-eclampsia** b	217 (7.1)	20 (3.5)	994 (1.3)	<0.001	435 (7.0)	12 (0.2)	1305 (1.6)	<0.001
**Cardiac/renal disease** b	21 (0.7)	0 (0.0)	162 (0.2)	0.108	51 (0.8)	77 (1.2)	321 (0.4)	<0.001
**Respiratory disease** b	11 (0.4)	3 (0.5)	377 (0.5)	0.593	41 (0.7)	100 (1.6)	360 (0.4)	<0.001
**Suspected fetal growth impairment** b	45 (1.5)	6 (1.1)	393 (0.5)	0.001	236 (3.8)	40 (0.6)	506 (0.6)	<0.001
**Diabetes** b	32 (1.0)	4 (0.7)	240 (0.3)	<0.001	85 (1.4)	75 (1.2)	352 (0.4)	<0.001
**Severe anaemia** b	41 (1.3)	6 (1.1)	494 (0.6)	0.032	56 (0.9)	11 (0.2)	318 (0.4)	0.002
**Vaginal bleeding** b	46 (1.5)	4 (0.7)	589 (0.8)	0.002	47 (0.8)	9 (0.1)	586 (0.7)	0.023
**HIV**	42 (1.4)	18 (3.2)	1298 (1.7)	0.130	27 (0.4)	4 (0.1)	324 (0.4)	0.002
Missing	78 (2.6)	19 (3.4)	2707 (3.5)		0 (0.0)	0 (0.0)	12 (0.0)	
**Malaria** b	207 (6.8)	45 (8.0)	7254 (9.4)	0.133	7 (0.1)	3 (0.0)	50 (0.1)	0.240
**Pyelonephritis or urinary infection** b	125 (4.1)	15 (4.4)	2752 (3.6)	0.666	79 (1.3)	40 (0.6)	967 (1.2)	0.281
**Delivery performed by** b								
Doctor	1,138 (37.3)	221 (39.2)	10581 (13.7)	<0.001	4,002 (64.5)	2,192 (35.0)	40911 (49.3)	0.001
Midwife/Nurse	1,727 (56.6)	316 (56.0)	60257 (78.3)		2,074 (33.4)	4,001 (63.8)	39027 (47.1)	
Other	184 (6.0)	27 (4.8)	6048 (7.9)		125 (2.0)	76 (1.2)	2984 (3.6)	
**Gestational age**								
<37 weeks	497 (16.3)	48 (8.5)	8586 (11.2)	<0.001	985 (15.9)	264 (4.2)	9330 (11.3)	<0.001
37–42 weeks	2,356 (77.2)	489 (86.7)	65530 (85.1)		5,164 (83.3)	5,996 (95.6)	72943 (88.0)	
>42 weeks	116 (3.8)	6 (1.1)	308 (0.4)		43 (0.7)	4 (0.1)	248 (0.3)	
Missing	84 (2.8)	21 (3.7)	2541 (3.3)		9 (0.1)	6 (0.1)	408 (0.5)	
**Fetal presentation** b								
Cephalic	2,881 (94.4)	537 (95.2)	73667 (95.7)	0.009	5,998 (96.7)	6,201 (98.9)	79265 (95.6)	<0.001
Breech	152 (5.0)	14 (2.5)	2615 (3.4)		151 (2.4)	41 (0.7)	3073 (3.7)	
Other	14 (0.5)	9 (1.6)	462 (0.6)		51 (0.8)	27 (0.4)	589 (0.7)	
**Sex** b								
Female	1,453 (47.9)	276 (49.4)	37,324 (48.8)	0.644	2,993 (48.3)	3,119 (49.7)	39.717 (47.9)	0.019
Male	1,579 (52.1)	283 (50.6)	39,179 (51.2)		3,203 (51.7)	3,149 (50.2)	43,193 (52.1)	

a Chi-square p-values are adjusted for survey design.

b Missing values below 1% were omitted.

**Table 2:** Facility characteristics by labour status in Africa and Asia.

	AFRICA				ASIA			
	Induction with medical indication	Elective induction	Spontaneous labour	Adjusted chi squareP value^a^	Induction with medical indication	Elective induction	Spontaneous labour	Adjusted chi squareP value^a^
	N, (%)	N, (%)	N, (%)		N, (%)	N, (%)	N, (%)	
**TOTAL**	**3,053**	**564**	**76,965**		**6,201**	**6,270**	**82,929**	
**Location** b				0.008				0.841
Urban	2,547 (84.6)	501 (88.8)	55,243 (72.2)		5,394 (87.1)	4,940 (78.9)	67,973 (82.3)	
Peri-urban	214 (7.0)	32 (5.7)	8,430 (11.0)		380 (6.1)	640 (10.2)	7,556 (9.1)	
Rural	285 (9.4)	31 (5.5)	12,845 (16.8)(		420 (6.8)	682 (10.9)	7,099 (8.6)	
**Level of facility** b								
Primary	214 (7.0)	41 (7.3)	12,201 (15.9)	0.004	0 (0.0)	0 (0.0)	0 (0.0)	0.078
Secondary	1,793 (58.7)	224 (39.7)	42,620 (55.4)		1,723 (27.8)	3,014 (48.1)	25,339 (30.6)	
Tertiary	895 (29.3)	223 (39.5)	15,629 (20.3)		4,346 (70.1)	3,208 (51.2)	52,043 (62.8)	
Other referral level	117 (3.8)	74 (13.1)	5,772 (7.5)		132 (2.1)	48 (0.8)	5,547 (6.7)	

a Chi-square p-values are adjusted for survey design.

b Missing values below 1% were omitted.

Rates of induction and their indications varied widely between regions and countries (Tables 3 and 4, Table S1 and S2). Although women not undergoing labour were excluded from this analysis, Table 3 includes these women to provide context. In Africa (average 4.4%), induction rates ranged from 1.4% in Niger to 6.8% in Algeria. Asian rates were generally higher (average 12.1%), ranging from 2.5% in Cambodia to 35.5% in Sri Lanka. Japan, the highest income country, had an induction rate of 19.0%. Induction without medical indication accounted for less than 2% of deliveries in all countries, except for Sri Lanka (27.8%), Japan (8.5%), India (3.6%) and Thailand (3.5%). In Africa, PROM (27.3%) was the most common indication, while in Asia 47.2% were elective. There were 448 women (166 in Africa and 282 in Asia) who had fetal distress as their only medical indication (data not shown). Amongst elective inductions in Africa and Asia, 36.0% and 32.1% were at <39 weeks gestation (data not shown).

**Table 3:** Labour and mode of delivery, by region.

	African countries	Asian countries
	N (%)	N (%)
**Number of facilities**	**131**	**122**
**Number of deliveries**	**83,437**	**109,101**
Spontaneous labour	76965 (92.3)	82929 (76.0)
Induction of labour	3700 (4.4)	13157 (12.1)
Induction with medical indication	3053 (3.7)	6201 (5.7)
Elective induction	564 (0.7)	6270 (5.7)
Indication missing	83 (0.1)	686 (0.6)
No Labour	2703 (3.2)	13013 (11.9)
Labour status missing	0 (0.0)	2 (0.0)

**Table 4:** Rates and indications for induction of labour, by region.

	African countries	Asian countries
	N, %^a^	N, %^a^
**All inductions**	**3,700**	**13,157**
Fetal death	305 (8.3)	359 (2.7)
Suspected fetal growth impairment	83 (2.2)	415 (3.2)
Fetal distress	693 (18.8)	558 (4.2)
Multiple pregnancy	101 (2.7)	105 (0.8)
Prelabour rupture of membranes	1,008 (27.3)	2,550 (19.4)
Chorioamnionitis	58 (1.6)	131 (1.0)
Vaginal bleeding	86 (2.3)	87 (0.7)
Pre-eclampsia/eclampsia	302 (8.2)	731 (5.6)
Gestational age > = 41 weeks	545 (14.8)	1,570 (11.9)
Elective induction	335 (9.1)	6213 (47.2)
Maternal Request	67 (1.8)	558 (4.2)
Any other obstetric complication	707 (19.2)	961 (7.3)
Any other medical complication	532 (14.4)	267 (2.0)

a Indications for induction were not mutually exclusive (ie: women could have >1 indication for induction). Percentages are calculated by (number of inductions for [indication]/all inductions) * 100.

Table 5 describes induction methods - oxytocin alone or oxytocin in combination with a non-drug method (sweeping of membranes, artificial rupture of membranes, mechanical methods or nipple stimulation) were the most common in Africa (45.9% and 20.2%) and Asia (31.5% and 28.2%). In Sri Lanka, induction was very common (35.5%) and the most popular methods were oxytocin plus a non-drug method (48.1%) and non-drug methods alone (24.2%) (Table S3). The use of misoprostol or another prostaglandin (either alone or in combination with other methods) averaged 15% of inductions across Africa and Asia. Induction success (inductions resulting in a vaginal birth) was 83.4% in Africa and 81.6% in Asia (Table 6). The most successful method was oxytocin only in Africa (86.1%) and oxytocin, misoprostol/other prostaglandin and a non-drug method in Asia (86.3%). Kenya, Uganda, China, Nepal, the Philippines, Thailand and Viet Nam had relatively lower success rates across all induction methods (Table S4).

**Table 5:** Mode of induction of labour, by region.

	African countries	Asian countries
	N, %	N,%
**All inductions**	**3,700**	**13,157**
Oxytocin only^a^	1697 (45.9)	4147 (31.5)
Misoprostol/other prostaglandins only^a^	542 (14.6)	2083 (15.8)
Non-drug methods only^a^^,^ b	131 (3.5)	1586 (12.1)
Oxytocin plus misoprostol/other prostaglandins only^a^	145 (3.9)	626 (4.8)
Oxytocin plus non-drug methods only^a^^,^ b	747 (20.2)	3711 (28.2)
Misoprostol/other prostaglandins plus non-drug methods^a^^,^ b	73 (2.0)	235 (1.8)
Oxytocin plus misoprostol/other prostaglandins plus non-drug methods^a^^,^ b	123 (3.3)	504 (3.8)
None/other^a^	229 (6.2)	262 (2.0)
Missing^a^	13 (0.4)	3 (0.0)

a percentage is calculated by (number of inductions by [mode]/all inductions) * 100.

b non-drug methods only includes any one or more of: sweeping membranes, artificial rupture of membranes, mechanical methods and nipple stimulation.

**Table 6:** Success of induction of labour, by region.

	African countries	Asian countries
	N, %	N,%
**All inductions**	**3,700**	**13,157**
Total success of induction (% having vaginal birth)^a^	3080 (83.4)	10739 (81.6)
Oxytocin only^b^	1458 (86.1)	3416 (82.4)
Misoprostol and/or other prostaglandins only^b^	448 (82.7)	1637 (78.6)
Non-drug methods only^b^^,^ c	108 (82.4)	1266 (78.6)
Oxytocin plus misoprostol and/or other prostaglandins only^b^	129 (89.0)	459 (73.4)
Oxytocin plus non-drug methods only^b^^,^ c	649 (86.9)	3181 (85.7)
Misoprostol and/or other prostaglandins plus non-drug methods^b^^,^ c	54 (74.0)	169 (71.9)
Oxytocin plus misoprostol/other prostaglandins plus non-drug methods^b^^,^ c	94 (76.4)	435 (86.3)
None/other^b^	129 (56.6)	173 (66.3)

a percentage calculated by (number of inductions resulting in vaginal delivery/all inductions) * 100.

b percentage calculated by (number of inductions by [mode] resulting in vaginal delivery/all inductions by [mode]) * 100.

c non-drug method only includes any one or more of: sweeping membranes, artificial rupture of membranes, mechanical methods and nipple stimulation.

**Figure 2:**
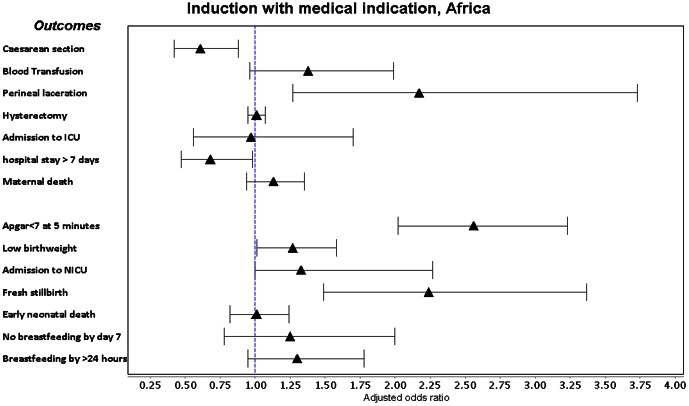
Forest plot of adjusted odds ratios and confidence intervals following medically indicated labour induction in Africa.

**Figure 3:**
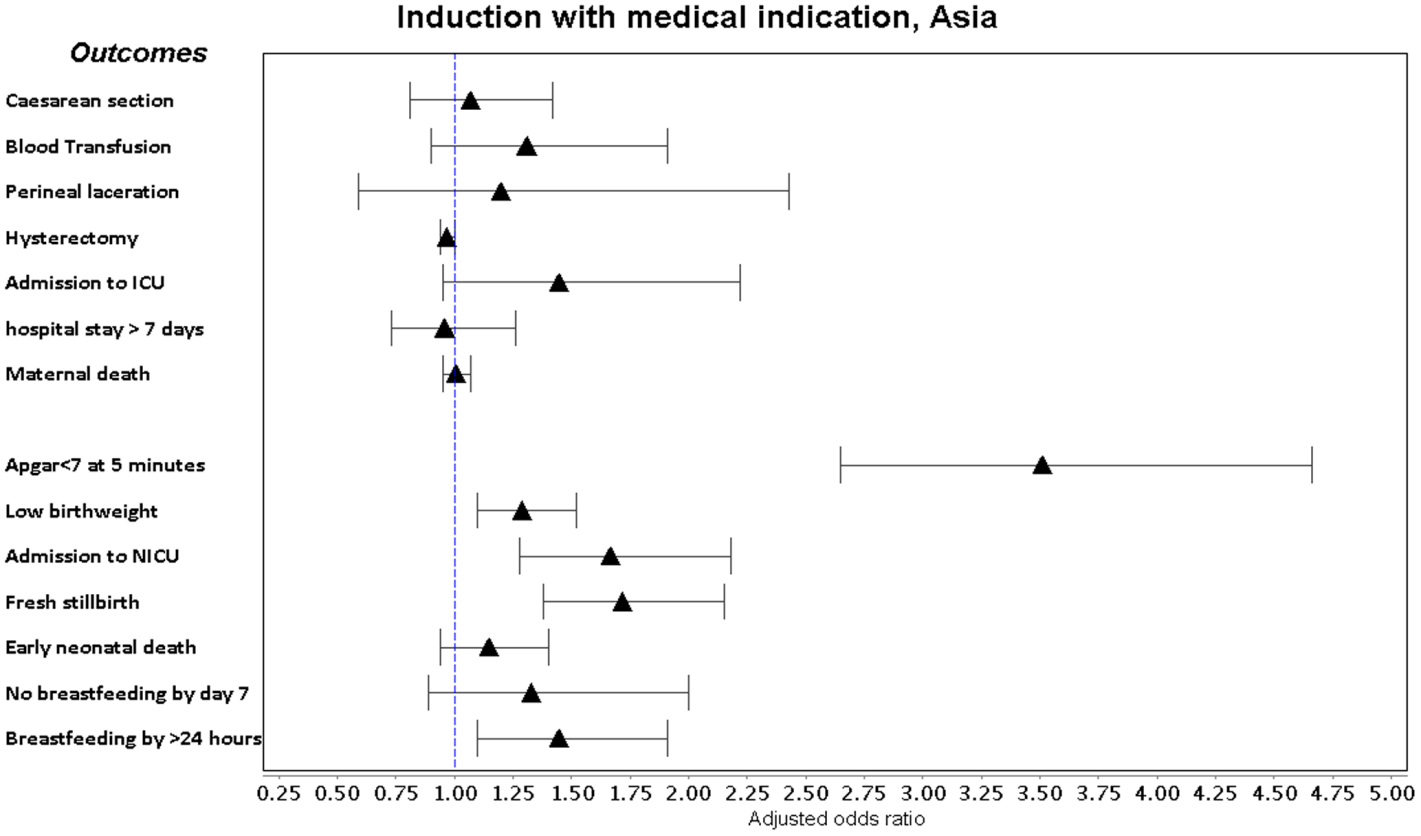
Forest plot of adjusted odds ratios and confidence intervals following medically indicated labour induction in Asia.

**Figure 4:**
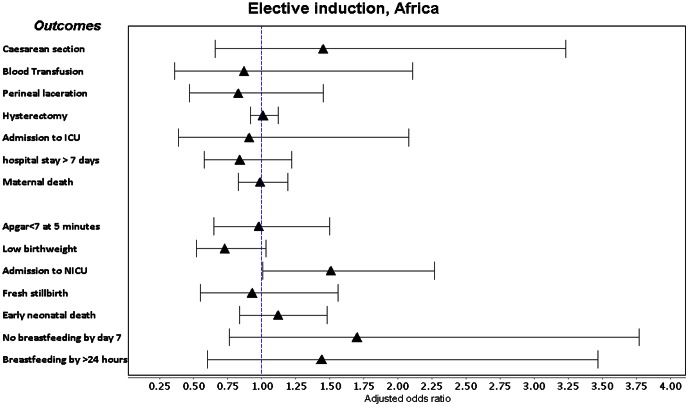
Forest plot of adjusted odds ratios and confidence intervals following elective induction in Africa.

**Figure 5:**
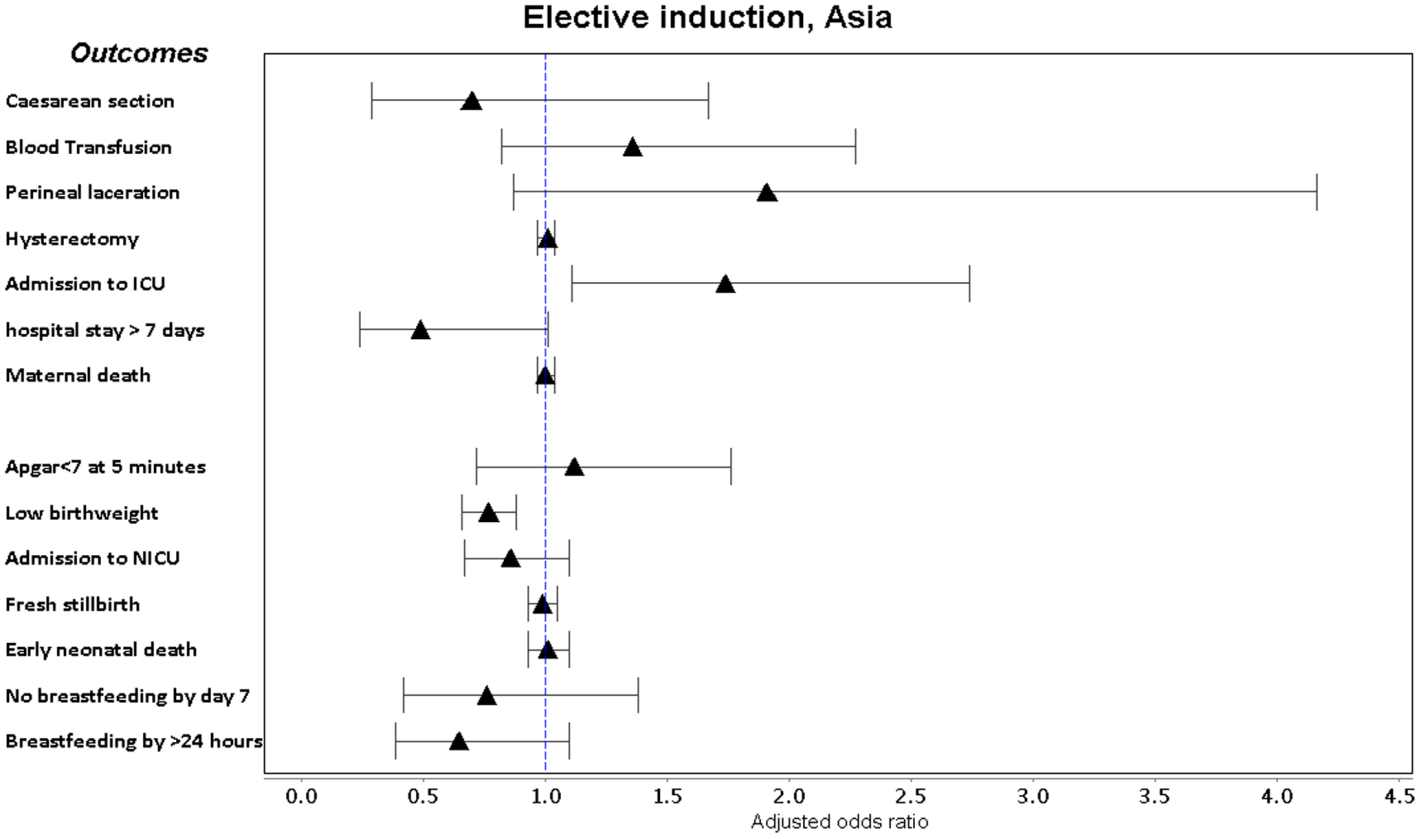
Forest plot of adjusted odds ratios and confidence intervals following elective induction in Asia.

Labour induction with a medical indication was associated with a consistent increase in adjusted odds of Apgar score <7 at 5 minutes, low birthweight, ICU admission and fresh stillbirth in both regions (Figures 2, 3, 4, 5, Table S5). It was also associated with increased adjusted odds of perineal laceration in Africa (Adj OR 2.17, 95% CI 1.27–3.73) and breastfeeding not commencing in the 24 hours after delivery in Asia (Adj OR 1.45, 95% CI 1.10–1.91). In Africa, the adjusted odds of caesarean section (Adj OR 0.61, 95% CI 0.42–0.88) and postpartum hospital stay over 7 days (Adj OR 0.68 95% CI 0.47–0.98) were decreased and the adjusted odds of hysterectomy in Asian countries (Adj OR 0.97, 95% CI 0.94–1.00) showed a borderline decrease. Induction without medical indication was associated with an increase in the adjusted odds of NICU admission in Africa (Adj OR 1.51 95% CI 1.01–2.27) and ICU admission in Asia (Adj OR 1.74 95% CI 1.11–2.74) and a decrease in the adjusted odds of low birthweight in Asia (Adj OR 0.77, 95% CI 0.66–0.88). We conducted a sensitivity analysis, excluding those women who had fetal distress as the only medical indication for labour induction, however crude and adjusted odds ratios did not alter in magnitude, direction or significance.

## Discussion

We aimed to describe the prevalence, indications, methods, success and outcomes of induction of labour in an international survey dataset of 192,538 deliveries in 253 facilities across 16 countries in Africa and Asia. Labour induction (both medically indicated and elective) was less common than reported for higher-income countries [1], [2], apart from a small group of Asian countries (Sri Lanka, Japan and India). Guerra et al hypothesized that fewer inductions in Latin America (11.4%) were due to low thresholds for caesarean delivery [4], [16] - while this is likely the case in some facilities African and Asian facilities, we believe in many resource-constrained settings the low induction rates probably represent a lack of familiarity and/or training, appropriate medications, monitoring capacity or necessary staff. WHO recommends only performing induction in facilities where caesarean section can be performed [3], [5], therefore increasing access to labour induction will require a concomitant improvement in access to safe caesarean section. Data from facilities in Sri Lanka were surprising - 35.5% of deliveries were induced, of which three-quarters were elective. One Sri Lankan hospital study reported a rate of 8.5% [1], [3], [5], [21] but no other literature is available to confirm this finding. Sri Lanka has made enormous progress in maternal health and 99% of deliveries now occur in hospitals [1], [2], [22]; access to elective induction may have consequently risen. Also, participating Sri Lankan institutions may have had an unusually high induction rate.

The pattern of medical indications for inductions in higher-income countries is evolving with changing demographics and pregnancy complications [6], [7]. In African and Asian facilities, prelabour rupture of membranes (27.3% and 19.3%) was the most common indication for medical induction, while prolonged pregnancy was 14.8% and 19.4% respectively. The medical indications for induction increase the risks of delivery itself, thus poorer maternal and perinatal outcomes are not unexpected. Higher attendance by doctors at induction deliveries may also reflect higher background risks, or perhaps doctors are managing inductions more often, carrying resource implications for upscaling induction in resource-constrained settings. Any form of induction in women with a previous caesarean section (2.2% in Africa and 1.6% in Asia) or a non-cephalic presentation (5.3% in Africa and 2.2% in Asia) suggest there is some degree of inappropriate patient selection taking place [8], [9], [23], potentially contributing to poorer outcomes.

Guerra et al reported an elective induction rate of 16.7% in Latin American facilities, while we found nearly 50% of inductions in Asian facilities were elective, driven by Sri Lanka (77.2%), Thailand (44.6%), Japan (41.0%), India (32.1%) and China (20.4%). This is more consistent with higher-income country practices [7]–[10] and may reflect pro-induction hospital policies or increasing maternal demand. Of particular note is that approximately one-third of elective inductions in African and Asian facilities occurred at <39 weeks. There is clear evidence that elective induction should not be performed before 39 weeks gestation, as perinatal outcomes are less favorable [2], [11]–[13], [24], [25]. While we did not find any increase in the risk of fetal or neonatal mortality, it is of concern that elective induction practices are putting neonates at unnecessary risk. Non-reassuring fetal status is a contraindication to induction of labour [8], [13], [14], [23] and we were surprised to find high rates of fetal distress as an indication for labour induction – 18.8% in Africa and 4.2% in Asia. Complications of induction (such as uterine hyperstimulation or cord prolapse) can cause fetal distress; as WHOGS data collectors reviewed medical records to collect individual data, it is possible that fetal distress was incorrectly classified as an indication rather than a consequence. Sensitivity analysis demonstrated that the 448 women induced for fetal distress alone did not significantly affect our results.

The WHO induction of labour guidelines recommended the use of vaginal or oral misoprostol (or other vaginal prostaglandins) as first-line induction agents in women without a previous caesarean section; intravenous oxytocin may be used if prostaglandins are not available [3], [15]. Prostaglandins have a reduced risk of not achieving vaginal birth within 24 hours and fewer caesarean births when compared to oxytocin alone [3]. Despite this, oxytocin alone was the most common method used in 10 of the 16 countries. The use of prostaglandins for induction is not yet widespread in these facilities despite its efficacy. This suggests that its use as an induction agent is not well known, compounded by the low availability of misoprostol in many lower-income countries (despite its many applications, long life and ease of administration and storage) [11], [16], [26], [27]. Success of induction was generally high, comparable to data from higher-income countries [7], [16], however success varied by country rather than method. As there are no consistent definitions of failure of induction [2], [11], it is unsurprising that country success rates differ. Universal criteria for determining failure of induction may improve success rates in certain contexts.

The adjusted odds of blood transfusion, maternal death, early neonatal death and commencement of breastfeeding by day 7 postpartum were not increased with medical indicated inductions. Odds of caesarean delivery were reduced in African women, supporting the findings of a Cochrane review that induced women (37 to 40 weeks) were less likely to have a caesarean section than women managed expectantly (RR 0.97, 95% CI 0.34 to 0.99), a difference that did not persist beyond 40 weeks [4], [17]. This must be interpreted with caution – the same was not found in Asian countries and the adjusted OR is in the opposite direction to the crude OR (OR 1.78, 95% CI 1.61–1.98), suggesting that while women induced for a medical indication have a greater overall odds of caesarean section, this is due to the effect of underlying confounders rather than induction itself. The Adj OR estimate is potentially confounded by factors we are unable to adjust for, such as cervical ripeness and the institution-specific threshold for failure of induction. It is however reassuring that no model displayed an increase in odds of caesarean section. The Cochrane review did not report on the effect of induction on length of stay, however our data suggests African women with a medically indicated induction had lower odds of hospital stays over 7 days. This carries hospital resource implications, an important consideration in resource-constrained settings. African women also had increased adjusted odds of perineal laceration, supporting the findings of Guerra et al in Latin American facilities (Adj RR 2.17, 95% CI 1.75–2.70). However, Asian women had a borderline decrease in the odds of hysterectomy, whereas Latin American women had approximately double the risk of hysterectomy.

Our data supports the Cochrane review findings that induction was associated with more NICU admissions and lower mean birthweight (after 42 weeks), as well as neonates requiring more postpartum care [4], [18], [19]. Guerra et al also found an increased risk of Apgar <7 at 5 minutes, very low birthweight, NICU admission and delayed breastfeeding associated with labour induction [16]. While the Cochrane review established that induction reduces perinatal mortality (RR 0.30, 95% CI 0.09–0.99) beyond 42 weeks gestation, only one stillbirth was reported in the seven trials included for this outcome. Fresh stillbirth is a relatively infrequent event in low mortality settings and has not been routinely considered as an outcome in other studies of induction. The increased odds of fresh stillbirth could be attributed to 1) higher maternal risk, due to morbidities for which induction was performed, 2) induction itself, 3) a combination of the stress of induction on fetuses born to higher-risk women, or 4) confounders we are unable to adjust for that contribute to stillbirth, such as intrapartum care. Given that labour induction without medical indication was not associated with a change in the odds of fresh stillbirth, we believe it more likely that the underlying increased fetal risk due to maternal morbidities was responsible.

The incidence of elective induction is increasing [6], [7] and clarifying the risk posed to mothers and babies in low- and middle-income countries is critical. While the majority of countries in our analysis had very low rates of elective induction, it was associated with increased odds of admissions to ICU (Africa) and NICU (Asia), but not increased odds of fetal or neonatal mortality. Guerra et al also found increased admission to ICU (ARR 2.90, 95% CI 1.24–6.78), but not NICU. One large study of elective induction in Scotland also found an increase in NICU admissions following elective induction [13]; whether this is only due to hospital policies following induction is unclear. Our findings will be strongly influenced by variable ICU/NICU availability and hospital policies and it is not possible to conclude this is due to increased morbidity. However, It is reassuring that elective induction was not associated with increased perinatal mortality. The reduced odds of low birthweight in Asia is likely due to an underlying bias in women induced electively, as induction is not causally related to birth weight.

This analysis has several strengths. It is the largest analysis of maternal and perinatal outcomes following induction in a largely low- and middle-income country dataset, addressing a significant literature gap. Separate analyses accounted for regional differences. Our analysis included a statistically useful number of rare outcomes, such as maternal, fetal and early neonatal deaths that allowed exploration of induction practices and outcomes in real world settings. However, this dataset did not record information on smoking, family history, race and other variables that are possible confounders. We also lacked information on Bishop scores, use of ripening agents, length of labour and doses or side effects of induction agents. The WHOGS data was collected from facilities with the capacity to perform caesarean sections – it is therefore not representative of countries or of all facilities, particularly in community settings. It is fair to assume that access to induction and caesarean is lower outside of similar facilities in these countries. However, we believe these results are generalizable to similar institutions and in many countries this data will be the best available on the use and consequences of induction. Future research should consider physician and patient factors contributing to the elective induction rate, cost-effectiveness in lower-income countries and induction practices at the level of smaller facilities and the community.

## Conclusion

Induction of labour (indicated and elective) is generally less common in lower-income than higher-income countries. Notable exceptions (such as Sri Lanka and India) suggest that scaling up induction availability in resource-constrained settings is possible. Despite strong evidence on the use of misoprostol and other prostaglandins, there is widespread use of oxytocin alone as an induction agent. While medically indicated inductions increase the odds of several adverse neonatal outcomes, this was likely influenced by higher baseline maternal risk. It was also associated with reduced odds of caesarean section and postpartum stay over 7 days in Africa and hysterectomy in Asia. Elective induction was not associated with increased odds of maternal, fetal or perinatal mortality, however one-third of elective inductions occurred at <39 weeks gestation. NICU admission in Africa and maternal ICU admission in Asia were significantly higher following elective induction which may have resource implications, however the risk of other adverse outcomes were not significantly higher.

## Supporting Information

Table S1 Labour and mode of delivery, by country.
(DOCX)

Table S2 Rates and indications for induction of labour, by country.
(DOCX)

Table S3 Mode of induction of labour, by country.
(DOCX)

Table S4 Success of induction of labour, by country.
(DOCX)

Table S5 Maternal and perinatal outcomes following induction of labour in Africa and Asia.
(DOCX)
