# Supplementary material for: Patterns and Outcomes of Induction of Labour in Africa and Asia: A Secondary Analysis of the WHO Global Survey on Maternal and Neonatal Health
Source: PLoS One. 2013 Jun 3;8(6):e65612. doi: 10.1371/journal.pone.0065612 (PMC3670838; doi:10.1371/journal.pone.0065612)
Supplement: Table S1 — Labour and mode of delivery, by country. (DOCX) [file pone.0065612.s001.docx]

**Table S1**

|  | **AFRICA** | | | | | | | **ASIA** | | | | | | | | |
| --- | --- | --- | --- | --- | --- | --- | --- | --- | --- | --- | --- | --- | --- | --- | --- | --- |
|  | **Algeria** | **Angola** | **DR Congo** | **Kenya** | **Niger** | **Nigeria** | **Uganda** | **Cambodia** | **China** | **India** | **Japan** | **Nepal** | **Philippines** | **Sri Lanka** | **Thailand** | **Viet Nam** |
| **Number of facilities** | **18** | **20** | **21** | **20** | **11** | **21** | **20** | **5** | **21** | **20** | **10** | **8** | **17** | **14** | **12** | **15** |
| **Number of deliveries** | **15,889** | **6,432** | **9,013** | **20,343** | **8,435** | **9,206** | **14,119** | **5,642** | **14,709** | **24,978** | **3,356** | **8,577** | **13,432** | **15,157** | **9,838** | **13,412** |
| Spontaneous labour | 13795 (86.8) | 6003 (93.5) | 8404 (93.3) | 18813 (92.6) | 8279 (98.2) | 8249 (98.8) | 13422 (95.2) | 5348 (94.8) | 9393 (63.9) | 20838 (83.4) | 2215 (66.0) | 7314 (85.3) | 11672 (86.9) | 6523 (43.0) | 7622 (77.5) | 12004 (89.5) |
| Induction of labour | 1073 (6.8) | 322 (5.0) | 462 (5.1) | 792 (3.9) | 118 (1.4) | 577 (6.3) | 356 (2.5) | 142  (2.5) | 937 (6.4) | 3192 (12.8) | 639 (19.0) | 702 (8.2) | 582  (4.3) | 5384 (35.5) | 814  (8.3) | 765 (5.7) |
| Induction with medical indication | 1017 (6.4) | 280 (4.4) | 380 (4.2) | 624 (3.1) | 7  (0.1) | 470 (5.1) | 221 (1.6) | 137  (2.4) | 569 (3.9) | 2121 (8.5) | 303 (9.0) | 594 (6.9) | 474  (3.5) | 927 (6.1) | 435  (4.4) | 641 (4.8) |
| Elective induction | 41  (0.3) | 36  (0.6) | 76  (0.8) | 139 (0.7) | 61  (0.7) | 96  (1.0) | 126 (0.9) | 2  (0.0) | 278 (1.9) | 900 (3.6) | 285 (8.5) | 71  (0.8) | 81  (0.6) | 4220 (27.8) | 348  (3.5) | 85  (0.6) |
| Indication missing | 15  (0.1) | 6  (0.1) | 6  (0.1) | 29  (0.1) | 50  (0.6) | 11  (0.1) | 9  (0.1) | 3  (0.1) | 90  (0.6) | 171 (0.7) | 51  (1.5) | 37  (0.4) | 27  (0.2) | 237 (1.6) | 31  (0.3) | 39  (0.3) |
| No Labour | 1019 (6.4) | 97  (1.5) | 142 (1.6) | 720 (3.5) | 38  (0.5) | 364 (4.0) | 323 (2.3) | 152  (2.7) | 4379 (29.8) | 948 (3.8) | 501 (14.9) | 561 (6.5) | 3250 (21.4) | 3250 (21.4) | 1401 (14.2) | 643 (4.8) |
| Labour status missing | 0  (0.0) | 0  (0.0) | 0  (0.0) | 0  (0.0) | 0  (0.0) | 0  (0.0) | 0  (0.0) | 0  (0.0) | 0  (0.0) | 0  (0.0) | 1  (0.0) | 0  (0.0) | 0  (0.0) | 0  (0.0) | 1  (0.0) | 0  (0.0) |
