# Supplementary material for: Patterns and Outcomes of Induction of Labour in Africa and Asia: A Secondary Analysis of the WHO Global Survey on Maternal and Neonatal Health
Source: PLoS One. 2013 Jun 3;8(6):e65612. doi: 10.1371/journal.pone.0065612 (PMC3670838; doi:10.1371/journal.pone.0065612)
Supplement: Table S2 — Rates and indications for induction of labour, by country. (DOCX) [file pone.0065612.s002.docx]

**Table S2**

|  | **AFRICA** | | | | | | | **ASIA** | | | | | | | | |
| --- | --- | --- | --- | --- | --- | --- | --- | --- | --- | --- | --- | --- | --- | --- | --- | --- |
|  | **Algeria** | **Angola** | **DR Congo** | **Kenya** | **Niger** | **Nigeria** | **Uganda** | **Cambodia** | **China** | **India** | **Japan** | **Nepal** | **Philippines** | **Sri Lanka** | **Thailand** | **Viet Nam** |
|  | N, % | N, % | N, % | N, % | N, % | N, % | N, % | N, % | N, % | N, % | N, % | N, % | N, % | N, % | N, % | N, % |
| **All inductions** | **1073** | **322** | **462** | **792** | **118** | **577** | **356** | **142** | **937** | **3192** | **639** | **702** | **582** | **5384** | **814** | **765** |
| **Fetal death** | 28  (2.6) | 30  (9.3) | 43  (9.3) | 67  (8.5) | 4  (3.4) | 74 (12.8) | 38 (10.7) | 27  (19.0) | 21  (2.2) | 200 (6.3) | 6  (0.9) | 27  (3.8) | 18  (3.1) | 36  (0.7) | 15  (1.8) | 9  (1.2) |
| **Suspected fetal growth impairment** | 28  (2.6) | 14  (4.4) | 6  (1.3) | 21  (2.7) | 0  (0.0) | 13  (2.3) | 1  (0.3) | 11  (7.7) | 9  (1.0) | 238 (7.5) | 39  (6.1) | 15  (2.1) | 13  (2.2) | 69  (1.3) | 10  (1.2) | 11  (1.4) |
| **Fetal distress** | 494 (46.0) | 95 (29.7) | 20  (4.3) | 28  (3.5) | 19 (16.1) | 34  (5.9) | 3  (0.8) | 11  (7.7) | 15  (1.6) | 327 (10.2) | 11  (1.7) | 58  (8.3) | 57  (9.8) | 24  (0.4) | 49  (6.0) | 6  (0.8) |
| **Multiple pregnancy** | 25  (2.3) | 18  (5.7) | 7  (1.5) | 12  (1.5) | 9  (7.6) | 22  (3.8) | 8  (2.2) | 5  (3.5) | 6  (0.6) | 50 (1.6) | 6  (0.9) | 12  (1.7) | 4  (0.7) | 14  (0.3) | 4  (0.5) | 4  (0.5) |
| **Prelabour rupture of membranes** | 355 (33.1) | 145 (45.3) | 170 (36.9) | 151 (19.1) | 10  (8.5) | 104 (18.1) | 73 (20.6) | 32  (22.5) | 257 (27.4) | 785 (24.6) | 119 (18.6) | 157 (22.4) | 152  (26.1) | 437 (8.1) | 157 (19.3) | 454 (59.3) |
| **Chorioamnionitis** | 18  (1.7) | 8  (2.5) | 22  (4.8) | 6  (0.8) | 0  (0.0) | 4  (0.7) | 0  (0.0) | 0  (0.0) | 1  (0.1) | 65  (2.0) | 4  (0.6) | 0  (0.0) | 5  (0.9) | 4  (0.1) | 4  (0.5) | 48  (6.3) |
| **Vaginal bleeding** | 20  (1.9) | 7  (2.2) | 16  (3.5) | 12  (1.5) | 3  (2.5) | 19  (3.3) | 9  (2.5) | 3  (2.1) | 2  (0.2) | 43  (1.3) | 2  (0.3) | 3  (0.4) | 8  (1.4) | 6  (0.1) | 13  (1.6) | 7  (0.9) |
| **Pre-eclampsia/ eclampsia** | 44  (4.1) | 7  (2.2) | 53 (11.5) | 92 (11.7) | 2  (1.7) | 46  (8.0) | 58 (16.4) | 38  (26.8) | 14  (1.5) | 446 (14.0) | 70 (11.0) | 23  (3.3) | 48  (8.2) | 47  (0.9) | 39  (4.8) | 6  (0.8) |
| **Gestational age >=41 weeks** | 131 (12.2) | 6  (1.9) | 59 (12.8) | 170 (21.5) | 161 (22.9) | 126 (21.9) | 43 (12.1) | 17  (12.0) | 247 (26.4) | 421 (13.2) | 13  (2.0) | 161 (22.9) | 143  (24.6) | 355 (6.6) | 89  (10.9) | 124 (16.2) |
| **Elective induction** | 28  (2.6) | 0  (0.0) | 70 (15.2) | 96 (12.2) | 39 (33.1) | 57  (9.9) | 45 (12.7) | 2  (1.4) | 191 (20.4) | 1026 (32.1) | 262 (41.0) | 49  (7.0) | 64  (11.0) | 4157 (77.2) | 363 (44.6) | 99 (12.9) |
| **Maternal Request** | 26  (2.4) | 0  (0.0) | 7  (1.5) | 12  (1.5) | 11  (9.3) | 4  (0.7) | 7  (2.0) | 3  (2.1) | 190 (20.3) | 8  (0.3) | 79 (12.4) | 47  (6.7) | 8  (1.4) | 193 (3.6) | 11  (1.4) | 19  (2.5) |
| **Any other obstetric complication** | 452 (42.1) | 2  (0.6) | 38 (8.2) | 128 (16.2) | 15 (12.7) | 52  (9.0) | 20  (5.6) | 29  (20.4) | 101 (10.8) | 116 (3.6) | 118 (18.5) | 222 (31.6) | 96  (16.5) | 124 (2.3) | 104 (12.8) | 20  (5.6) |
| **Any other medical complication** | 435 (40.5) | 25  (7.8) | 10  (2.2) | 13  (1.6) | 4  (3.4) | 36  (6.2) | 9  (2.5) | 5  (3.5) | 23  (2.5) | 81  (2.5) | 7  (1.1) | 13  (1.9) | 23  (4.0) | 88  (1.6) | 16  (2.0) | 11  (1.4) |

Indications for induction were not mutually exclusive (ie: women could have >1 indication for induction). Percentages are calculated by (number of inductions for [indication] / all inductions) * 100
