# Supplementary material for: Patterns and Outcomes of Induction of Labour in Africa and Asia: A Secondary Analysis of the WHO Global Survey on Maternal and Neonatal Health
Source: PLoS One. 2013 Jun 3;8(6):e65612. doi: 10.1371/journal.pone.0065612 (PMC3670838; doi:10.1371/journal.pone.0065612)
Supplement: Table S3 — Mode of induction of labour, by country. (DOCX) [file pone.0065612.s003.docx]

**Table S3**

|  | **AFRICA** | | | | | | | **ASIAN** | | | | | | | | |
| --- | --- | --- | --- | --- | --- | --- | --- | --- | --- | --- | --- | --- | --- | --- | --- | --- |
|  | **Algeria** | **Angola** | **DR Congo** | **Kenya** | **Niger** | **Nigeria** | **Uganda** | **Cambodia** | **China** | **India** | **Japan** | **Nepal** | **Philippines** | **Sri Lanka** | **Thailand** | **Viet Nam** |
|  | N, % | N, % | N, % | N, % | N, % | N, % | N, % | N, % | N, % | N, % | N, % | N, % | N, % | N, % | N, % | N, % |
| **All inductions** | **1073** | **322** | **462** | **792** | **118** | **577** | **356** | **142** | **937** | **3192** | **639** | **702** | **582** | **5384** | **814** | **765** |
| **Oxytocin only^a^** | 514 (47.9) | 249 (77.3) | 266 (57.6) | 207 (26.1) | 29 (24.6) | 286 (49.6) | 146 (41.0) | 49 (34.5) | 266 (64.4) | 754 (23.6) | 203 (31.8) | 405 (57.7) | 196 (33.7) | 1097 (20.4) | 441 (54.2) | 399 (52.2) |
| **Misoprostol/other prostaglandins only^a^** | 16 (1.5) | 33 (10.2) | 111 (24.0) | 201 (25.4) | 0 (0.0) | 88 (15.3) | 93 (26.1) | 73 (51.4) | 130 (13.9) | 1348 (42.2) | 48 (7.5) | 168 (23.9) | 30 (5.2) | 135 (2.5) | 24 (2.9) | 127 (16.6) |
| **Non-drug methods only^a,b^** | 55 (5.1) | 19 (5.9) | 16 (3.5) | 23 (2.9) | 7 (5.9) | 11 (1.9) | 0  (0.0) | 2 (1.4) | 17 (1.8) | 62 (1.9) | 40 (6.3) | 11 (1.6) | 28 (4.8) | 1301 (24.2) | 86 (10.6) | 39 (5.1) |
| **Oxytocin plus misoprostol/other prostaglandins only^a^** | 11 (1.0) | 4  (1.2) | 50 (10.8) | 62 (7.8) | 0  (0.0) | 13 (2.3) | 5  (1.4) | 9  (6.3) | 34 (3.6) | 370 (11.6) | 26 (4.1) | 81 (11.5) | 25 (4.3) | 38 (0.7) | 15 (1.8) | 28 (3.7) |
| **Oxytocin plus non-drug methods only^a,b^** | 458 (42.7) | 4  (1.2) | 9  (1.9) | 111 (14.0) | 64 (54.2) | 97 (16.8) | 4  (1.1) | 7  (4.9) | 118 (12.6) | 218 (6.8) | 141 (22.1) | 19 (2.7) | 217 (37.3) | 2591 (48.1) | 235 (28.9) | 165 (21.6) |
| **Misoprostol/other prostaglandins plus non-drug methods^a,b^** | 8  (0.7) | 0  (0.0) | 5  (1.1) | 49 (6.2) | 1  (0.8) | 7  (1.2) | 3  (0.8) | 2  (1.4) | 11 (1.2) | 49 (1.5) | 33 (5.2) | 6  (0.9) | 13 (2.2) | 114 (2.1) | 5  (0.6) | 2  (0.3) |
| **Oxytocin plus misoprostol/other prostaglandins plus non-drug methods^a,b^** | 6  (0.6) | 0  (0.0) | 1  (0.2) | 98 (12.4) | 0  (0.0) | 17 (2.9) | 1  (0.3) | 0  (0.0) | 11 (1.2) | 250 (7.8) | 144 (22.5) | 1  (0.1) | 29 (5.0) | 66 (1.2) | 3  (0.4) | 0  (0.0) |
| **None/other^a^** | 5  (0.5) | 10 (3.1) | 3  (0.6) | 38 (4.8) | 17 (14.4) | 57 (9.9) | 99 (27.8) | 0  (0.0) | 13 (1.4) | 141 (4.4) | 1  (0.2) | 11 (1.6) | 44 (7.6) | 42 (0.8) | 5  (0.6) | 5  (0.7) |
| **Missing^a^** | 0  (0.0) | 3  (0.9) | 1  (0.2) | 3  (0.4) | 0  (0.0) | 1  (0.2) | 5  (1.4) | 0  (0.0) | 0  (0.0) | 0  (0.0) | 3  (0.5) | 0  (0.0) | 0  (0.0) | 0  (0.0) | 0  (0.0) | 0  (0.0) |

^a^ percentage is calculated by (number of inductions by [mode] / all inductions) * 100

^b^ non-drug methods only includes any one or more of: sweeping membranes, artificial rupture of membranes, mechanical methods and nipple stimulation
