# Supplementary material for: Patterns and Outcomes of Induction of Labour in Africa and Asia: A Secondary Analysis of the WHO Global Survey on Maternal and Neonatal Health
Source: PLoS One. 2013 Jun 3;8(6):e65612. doi: 10.1371/journal.pone.0065612 (PMC3670838; doi:10.1371/journal.pone.0065612)
Supplement: Table S4 — Success of induction of labour, by country. (DOCX) [file pone.0065612.s004.docx]

**Table S4**

|  | **AFRICA** | | | | | | | **ASIA** | | | | | | | | |
| --- | --- | --- | --- | --- | --- | --- | --- | --- | --- | --- | --- | --- | --- | --- | --- | --- |
|  | **Algeria** | **Angola** | **DR Congo** | **Kenya** | **Niger** | **Nigeria** | **Uganda** | **Cambodia** | **China** | **India** | **Japan** | **Nepal** | **Philippines** | **Sri Lanka** | **Thailand** | **Viet Nam** |
|  | N, % | N, % | N, % | N, % | N, % | N, % | N, % | N, % | N, % | N, % | N, % | N, % | N, % | N, % | N, % | N, % |
| **All inductions** | **1073** | **322** | **462** | **792** | **118** | **577** | **356** | **142** | **937** | **3192** | **639** | **702** | **582** | **5384** | **814** | **765** |
| **Total success of induction (% having vaginal birth)^a^** | 949 (88.4) | 306 (95.0) | 407 (88.1) | 610 (77.0) | 106 (89.8) | 498 (86.3) | 204 (57.3) | 128  (90.1) | 636 (67.9) | 2676 (83.8) | 570 (89.2) | 561 (79.9) | 463  (79.6) | 4563 (84.8) | 559 (68.7) | 583 (76.2) |
| **Oxytocin only^b^** | 440 (85.6) | 237 (96.0) | 235 (88.3) | 176 (85.0) | 26 (89.7) | 257 (90.2) | 87 (60.0) | 44  (89.8) | 415 (68.8) | 700 (92.8) | 180 (88.7) | 345 (85.4) | 157  (80.1) | 986 (89.9) | 276 (62.6) | 313 (78.4) |
| **Misoprostol and/or other prostaglandins only^b^** | 15 (93.8) | 33 (100) | 100 (90.1) | 145 (72.1) | 0  (0.0) | 80 (90.9) | 75 (80.6) | 67  (91.8) | 88 (67.7) | 1085 (80.5) | 44 (91.7) | 121 (72.0) | 16  (53.3) | 95 (70.4) | 16 (66.7) | 105 (82.7) |
| **Non-drug methods only^b,c^** | 47 (85.5) | 19 (100) | 8 (50.0) | 18 (78.3) | 7 (100) | 9 (81.8) | 0  (0.0) | 1  (50.0) | 11 (64.7) | 48 (77.4) | 35 (87.5) | 5 (45.5) | 21  (75.0) | 1059 (81.4) | 67 (77.9) | 19 (48.7) |
| **Oxytocin plus misoprostol and/or other prostaglandins only^b^** | 10 (90.9) | 4 (100) | 48 (96.0) | 52 (83.9) | 0  (0.0) | 10 (76.9) | 5 (100) | 8  (88.9) | 15 (44.1) | 293 (79.2) | 20 (76.9) | 59 (73.8) | 10  (40.0) | 31 (81.6) | 8  (53.3) | 15 (53.6) |
| **Oxytocin plus non-drug methods only^b,c^** | 421 (91.9) | 3 (75.0) | 7 (77.8) | 84 (75.7) | 58 (90.6) | 73 (75.3) | 3 (75.0) | 6  (85.7) | 82 (69.5) | 199 (91.3) | 132 (93.6) | 17 (89.5) | 193  (88.9) | 2239 (86.4) | 184 (78.3) | 129 (78.2) |
| **Misoprostol and/or other prostaglandins plus non-drug methods^b,c^** | 6 (75.0) | 0  (0.0) | 4 (80.0) | 33 (67.3) | 1 (100) | 7  (100) | 3  (100) | 2  (100) | 9 (81.8) | 35 (71.4) | 25 (75.8) | 4 (66.7) | 10  (76.9) | 80 (70.2) | 4  (80.0) | 0  (0.0) |
| **Oxytocin plus misoprostol/other prostaglandins plus non-drug methods^b,c^** | 5 (83.3) | 0  (0.0) | 1 (100) | 74 (75.5) | 0  (0.0) | 13 (76.5) | 1  (100) | 0  (0.0) | 8 (72.7) | 216 (86.4) | 130 (90.3) | 1 (100) | 22  (75.9) | 56 (84.8) | 2  (66.7) | 0  (0.0) |
| **None/other^b^** | 5  (100) | 8 (80.0) | 3 (100) | 26 (68.4) | 14 (82.4) | 48 (85.7) | 25 (25.3) | 0  (0.0) | 8 (61.5) | 100 (71.4) | 1 (100) | 9 (81.8) | 34  (77.3) | 17 (40.5) | 2  (40.0) | 2 (40.0) |

^a^ percentage calculated by (number of inductions resulting in vaginal delivery / all inductions) * 100

^b^ percentage calculated (number of inductions by [mode] resulting in vaginal delivery / all inductions by [mode]) * 100

**^c^** non-drug method only includes any one or more of: sweeping membranes, artificial rupture of membranes, mechanical methods and nipple stimulation
