# Supplementary material for: Patterns and Outcomes of Induction of Labour in Africa and Asia: A Secondary Analysis of the WHO Global Survey on Maternal and Neonatal Health
Source: PLoS One. 2013 Jun 3;8(6):e65612. doi: 10.1371/journal.pone.0065612 (PMC3670838; doi:10.1371/journal.pone.0065612)
Supplement: Table S5 — Maternal and perinatal outcomes following induction of labour in Africa and Asia. (DOCX) [file pone.0065612.s005.docx]

**Table S5**

|  | | **African countries** | | | **Asian countries** | | |
| --- | --- | --- | --- | --- | --- | --- | --- |
|  |  | **n/N, (%)** | **Crude OR**  **(95% CI)** | **Adjusted OR**  **(95% CI)** | **n/N, (%)** | **Crude OR**  **(95% CI)** | **Adjusted OR**  **(95% CI)** |
| **Caesarean section** | Spontaneous | 7245/76965 (9.4) | Ref | Ref | 14899/82929 (18.0) | Ref | Ref |
|  | Induction with indication | 455/3053 (14.9) | 1.78 (1.61 - 1.98) | 0.61 (0.42 - 0.88) | 1409/6201 (22.7) | 1.37 (1.29 - 1.46) | 1.07 (0.81 - 1.42) |
|  | Induction without indication | 148/564 (26.2) | 3.56 (2.94 - 4.31) | 1.45 (0.66 - 3.23) | 861/6270 (13.7) | 0.74 (0.69 - 0.80) | 0.70 (0.29 - 1.67) |
| **Blood Transfusion** | Spontaneous | 4/134 (3.0) | Ref | Ref | 1214/82929 (98.5) | Ref | Ref |
|  | Induction with indication | 97/3050 (3.2) | 2.01 (1.63 - 2.49) | 1.38 (0.96 - 1.99) | 159/6201 (2.6) | 1.77 (1.50 - 2.09) | 1.31 (0.90 - 1.91) |
|  | Induction without indication | 13/563 (2.3) | 1.45 (0.83 - 2.52) | 0.87 (0.36 - 2.11) | 72/6270 (1.1) | 0.78 (0.62 - 0.99) | 1.36 (0.82 - 2.27) |
| **Perineal laceration** | Spontaneous | 1554/76898 (2.0) | Ref | Ref | 1810/82929 (2.2) | Ref | Ref |
|  | Induction with indication | 136/3052 (4.5) | 2.26 (1.89 - 2.71) | 2.17 (1.27 - 3.73) | 57/6201 (0.9) | 0.42 (0.32 - 0.54) | 1.20 (0.59 - 2.43) |
|  | Induction without indication | 8/564 (1.4) | 0.70 (0.35 - 1.40) | 0.83 (0.47 - 1.45) | 44/6270 (0.7) | 0.32 (0.24 - 0.43) | 1.91 (0.87 - 4.16) |
| **Hysterectomy** | Spontaneous | 65/76931 (0.1) | Ref | Ref | 71/82929 (0.1) | Ref | Ref |
|  | Induction with indication | 6/3053 (0.2) | 2.33 (1.01 - 5.38) | 1.01 (0.95 - 1.07) | 5/6201 (0.1) | 0.94 (0.38 - 2.33) | 0.97 (0.94 - 1.00) |
|  | Induction without indication | 1/564 (0.2) | 2.10 (0.29 - 15.16) | 1.01 (0.92 - 1.12) | 7/6270 (0.1) | 1.30 (0.60 - 2.84) | 1.01 (0.97 - 1.04) |
| **Admission to ICU** | Spontaneous | 1988/76929 (2.6) | Ref | Ref | 1695/82929 (2.0) | Ref | Ref |
|  | Induction with indication | 181/3052 (5.9) | 2.38 (2.03 - 2.78) | 0.97 (0.56 - 1.70) | 180/6201 (2.9) | 1.43 (1.23 - 1.68) | 1.45 (0.95 - 2.22) |
|  | Induction without indication | 13/564 (2.3) | 0.89 (0.51 - 1.54) | 0.91 (0.39 - 2.08) | 47/6270 (0.7) | 0.36 (0.27 - 0.48) | 1.74 (1.11 - 2.74) |
| **Hospital stay > 7 days** | Spontaneous | 5086/76965 (6.6) | Ref | Ref | 6000/82929 (7.2) | Ref | Ref |
|  | Induction with indication | 205/3053 (6.7) | 1.02 (0.88 - 1.18) | 0.68 (0.47 - 0.98) | 691 / 6201 (11.1) | 1.61 (1.48 - 1.75) | 0.96 (0.73 - 1.26) |
|  | Induction without indication | 51/564 (9.0) | 1.41 (1.06 - 1.88) | 0.84 (0.58 - 1.22) | 308 / 6270 (4.9) | 0.66 (0.59 - 0.75) | 0.49 (0.24 - 1.01) |
| **Maternal death** | Spontaneous | 186/76965 (0.2) | Ref | Ref | 73/82929 (0.1) | Ref | Ref |
|  | Induction with indication | 34/3053 (1.1) | 4.64 (3.22 - 6.71) | 1.13 (0.94 - 1.35) | 13/6201 (0.2) | 2.40 (1.33 - 4.33) | 1.01 (0.95 - 1.07) |
|  | Induction without indication | 1/564 (0.2) | 0.74 (0.10 - 5.26) | 0.99 (0.83 - 1.19) | 2/6270 (0.0) | 0.36 (0.09 - 1.48) | 1.00 (0.97 - 1.04) |
| **Apgar score <7 at 5 minutes** | Spontaneous | 5360/76965 (7.0) | Ref | Ref | 3368/82929 (4.1) | Ref | Ref |
|  | Induction with indication | 602/3053 (19.7) | 3.27 (2.98 - 3.59) | 2.56 (2.02 - 3.23) | 675/6201 (10.9) | 2.89 (2.65 - 3.15) | 3.51 (2.65 - 4.66) |
|  | Induction without indication | 45/564 (8.0) | 1.17 (0.86 - 1.59) | 0.98 (0.65 - 1.50) | 87/6270 (1.4) | 0.33 (0.27 - 0.41) | 1.12 (0.72 - 1.76) |
| **Low birthweight** | Spontaneous | 7069/76965 (9.2) | Ref | Ref | 10973/82929 (13.2) | Ref | Ref |
|  | Induction with indication | 410/3053 (13.4) | 1.55 (1.39 - 1.72) | 1.27 (1.01 - 1.58) | 1306/6201 (21.1) | 1.75 (1.64 - 1.87) | 1.29 (1.10 - 1.52) |
|  | Induction without indication | 44/564 (7.8) | 0.84 (0.62 - 1.15) | 0.73 (0.52 - 1.03) | 659/6270 (10.5) | 0.77 (0.71 - 0.84) | 0.77 (0.66 - 0.88) |
| **Admission to NICU** | Spontaneous | 5325/76965 (6.9) | Ref | Ref | 7535/82929 (9.1) | Ref | Ref |
|  | Induction with indication | 396/3053 (13.0) | 2.26 (2.02 - 2.52) | 1.33 (1.00 - 2.27) | 842/6201 (13.6) | 1.69 (1.56 - 1.82) | 1.67 (1.28 - 2.18) |
|  | Induction without indication | 73/564 (12.9) | 2.04 (1.59 - 2.61) | 1.51 (1.01 - 2.27) | 405/6270 (6.5) | 0.68 (0.62 - 0.76) | 0.86 (0.67 - 1.10) |
| **Fresh stillbirth** | Spontaneous | 1581/76965 (2.1) | Ref | Ref | 795/82929 (1.0) | Ref | Ref |
|  | Induction with indication | 145/3053 (4.7) | 2.55 (2.14 - 3.04) | 2.24 (1.49 - 3.37) | 224/6201 (3.6) | 4.00 (3.44 - 4.65) | 1.72 (1.38 - 2.15) |
|  | Induction without indication | 11/564 (2.0) | 0.96 (0.53 - 1.75) | 0.93 (0.55 - 1.56) | 12/6270 (0.2) | 0.20 (0.11 - 0.35) | 0.99 (0.93 - 1.05) |
| **Early neonatal death** | Spontaneous | 835/76965 (1.1) | Ref | Ref | 542/82929 (0.7) | Ref | Ref |
|  | Induction with indication | 45/3053 (1.5) | 1.51 (1.12 - 2.04) | 1.01 (0.82 - 1.24) | 73/6201 (1.2) | 1.92 (1.51 - 2.46) | 1.15 (0.94 - 1.40) |
|  | Induction without indication | 9/564 (1.6) | 1.51 (0.78 - 2.92) | 1.12 (0.84 - 1.48) | 11/6270 (0.2) | 0.27 (0.15 - 0.48) | 1.01 (0.93 - 1.10) |
| **Breastfeeding not commenced by day 7** | Spontaneous | 1423/76965 (1.8) | Ref | Ref | 1861/82929 (2.2) | Ref | Ref |
|  | Induction with indication | 97/3053 (3.2) | 2.23 (1.81 - 2.77) | 1.25 (0.78 - 2.00) | 187/6201 (3.0) | 1.41 (1.21 - 1.64) | 1.33 (0.89 - 2.00) |
|  | Induction without indication | 12/564 (2.1) | 1.55 (0.85 - 2.77) | 1.70 (0.76 - 3.77) | 35/6270 (0.6) | 0.21 (0.15 - 0.29) | 0.76 (0.42 - 1.38) |
| **Breastfeeding not commenced by 24 hours** | Spontaneous | 2295/76965 (3.0) | Ref | Ref | 5449/82929 (6.6) | Ref | Ref |
|  | Induction with indication | 161/3053 (5.3) | 2.30 (1.94 - 2.72) | 1.30 (0.95 - 1.78) | 391/6201 (6.3) | 1.00 (0.90 - 1.12) | 1.45 (1.10 - 1.91) |
|  | Induction without indication | 29/564 (5.1) | 2.32 (1.57 - 3.42) | 1.44 (0.60 - 3.47) | 126/6270 (2.0) | 0.25 (0.21 - 0.30) | 0.65 (0.39 - 1.10) |

^a^Model adjusted for: maternal age (<18, 18-35, >35), marital status, years of maternal education (0, 1-4, 5-9, >=10), parity (0, 1 – 2, >=3), number of antenatal care visits (0, 1 – 3, >=4), maternal height (<145, 145 – 155, >155), previous caesarean section, prelabour rupture of membranes, pre-eclampsia, chronic hypertension, pregnancy-induced hypertension, cardiac/renal disease, respiratory disease, suspected fetal growth impairment, diabetes, severe anaemia, vaginal bleeding in the second half of pregnancy, HIV, malaria, gestational age (<37 weeks, 37 – 42 weeks, >42 weeks), urinary infection/pyelonephritis, fetal presentation (cephalic, breech or other), attendant at delivery (doctor, midwife/nurse, other) and facility (as a random effect)

^b^ Model also adjusted for mode of delivery (vaginal or caesarean section)

^c^ Model also adjusted for infant sex and congenital malformation
